# Editorial: The differentiation and reprogramming of tumor-associated myeloid cells and therapeutic strategies for cancer immunotherapy

**DOI:** 10.3389/fimmu.2026.2016743

**Published:** 2026-10-16

**Authors:** Paul Takam Kamga, Lionel Franz Poulin, Li Lily Wang

**Affiliations:** 1 Université Paris-Saclay, Laboratoire d’Imagerie Biomédicale Multimodale (BIOMAPS), INSERM UMR1281, CNRS UMR9011, Orsay, France; 2 Department of Respiratory Diseases and Thoracic Oncology, Cancer Institute AP-HP, Paris-Saclay University, Ambroise Paré Hospital, Boulogne-Billancourt, France; 3 Nantes Université, Univ Angers, CHU Nantes, INSERM, CNRS, Immunology and New Concepts in ImmunoTherapy, INCIT, UMR 1302/EMR6001, Nantes, France; 4 Department of Cancer Science, Cleveland Clinic Foundation, Cleveland, OH, United States; 5 Case Comprehensive Cancer Center, Cleveland, OH, United States

**Keywords:** cancer immunotherapy, inflammatory cytokine, metabolic reprogramming, myeloid derived suppressor cells (MDSCs), myeloid polarization, peptide conjugate, tumor-associated macrophages (TAM), tumor-associated myeloid cells

Immunotherapies have transformed the treatment of many cancers, yet most patients derive no durable benefit, in part because efficacy depends not only on the direct effects of these agents on immune effector cells, but also on their ability to reshape cellular and molecular interactions within the tumor microenvironment toward conditions that support antitumor immune responses (1, 2). Myeloid cells are central to that context. This heterogeneous compartment includes monocytes, tumor-associated macrophages (TAMs), dendritic cells, myeloid-derived suppressor cells (MDSCs), and granulocytic populations that normally support innate immune surveillance, antigen presentation, tissue homeostasis, and the development of adaptive immunity. Within tumors, however, these cells are differentiated or reprogrammed toward states that suppress CD8^+^ T cells and NK cell function, thereby reinforcing immunosuppressive networks and blunting the response to immunotherapy (3, 4). The remarkable plasticity of myeloid populations has emerged as a major determinant of antitumor immunity and therapeutic responses (5). The seven contributions to this Research Topic examine how tumor-associated myeloid cells are characterized, monitored, and therapeutically modulated. Together, they support the premise that the tumor-associated myeloid compartment is therapeutically reprogrammable and that context-specific myeloid reprogramming may improve the efficacy of cancer immunotherapy.

## 1 Myeloid states as dynamic determinants of therapeutic response

The shift of tumor-associated myeloid cells, particularly macrophages, toward immunosuppressive phenotypes is a key event in tumor progression and resistance to immunotherapy. These phenotypes result from chronic education by the tumor microenvironment, yet increasing evidence indicates that they are not irreversible and can be functionally redirected. Multiple signaling axes and soluble mediators critically regulate the recruitment, differentiation, and polarization of tumor-associated macrophages, including CSF-1/CSF1R, IL-34/CSF1R, CCL2/CCR2, IL-4/IL-13–STAT6, IL-6–JAK/STAT, and TGF-β signaling, each of which is therapeutically tractable (3–6). The M1/M2 terminology remains as a convenient functional shorthand, but TAMs occupy a continuum of transcriptional, metabolic, and spatially defined states that cannot be fully captured by a binary classification (4, 6, 7).

The contributions to this Research Topic show that the abundance of tumor-associated macrophages, together with their functional state and molecular signature, can provide relevant information about the efficacy of anticancer strategies, including immunotherapy. The study by Gu et al. identified CXCL2-positive macrophages as a distinct population enriched in patients with esophageal squamous cell carcinoma who responded to immunotherapy, and found that low intratumoral infiltration of these cells correlated with poor survival. In murine ESCC models, CXCL2 signaling promoted a more immune-activated macrophage phenotype through cytosolic calcium influx and enhanced anti-PD-1 activity. These findings support the concept that a defined macrophage state may serve as both a biomarker and a functional determinant of therapeutic response.

Beyond individual markers, myeloid reprogramming can be viewed as a broader remodeling of the myeloid landscape. Song et al. paired single-cell RNA sequencing with complementary tissue analyses in patients with intrahepatic cholangiocarcinoma receiving gemcitabine-cisplatin chemotherapy plus anti-PD-L1 therapy. Tumors achieving a pathological response displayed lower fractions of CCL18-positive and SPP1-positive macrophages, showed a shift from an M2-like immunosuppressive state toward a more inflammatory macrophage state, and exhibited enhanced CD8^+^ T-cell activation. These findings highlight the value of global characterization of the myeloid compartment for understanding treatment-associated immune remodeling and therapeutic response.

Myeloid remodeling is also temporally dynamic, and the clinical significance of myeloid cell states depends upon when in the course of treatment and disease context (7). In a prospective study of patients with glioblastoma receiving chemoradiotherapy, Sloan et al. observed an increase in circulating MDSCs toward the end of treatment among patients who were not alive two years after diagnosis, accompanied by a decrease in IFN-γ-producing CD8^+^ T cells. Baseline tumor myeloid characteristics did not distinguish these groups. These findings emphasize that treatment-induced immune changes can be clinically informative where pretreatment assessment alone cannot, supporting the value of longitudinal immune monitoring.

## 2 Therapeutic reprogramming of the myeloid compartment

Beyond their value as biomarkers, myeloid populations can themselves constitute therapeutic targets (8). Therapeutic modulation of tumor-associated myeloid cells may act through complementary mechanisms, including limiting the recruitment or survival of suppressive subsets, redirecting differentiation and activation states, and altering metabolic or transcriptional programs that sustain immunosuppression. One direct strategy is to interfere with the signaling pathways that control macrophage polarization, including the JAK/STAT pathway (6, 9).

Yeun et al. developed a peptide–drug conjugate, TAMpep-IP, which combined an M2-homing peptide with a STAT6-inhibitory peptide. In the CT26 murine colorectal cancer model, this peptide conjugate reduced STAT6 phosphorylation and downregulated M2-associated mediators such as CD206, Arg-1, TGF-β, and IL-13. Tumor growth was suppressed, with reduced infiltration of immunosuppressive macrophages and enhanced granzyme B-positive CD8^+^ T-cell responses. The study therefore provides a mechanistic demonstration that direct interference with macrophage polarization can translate into enhanced antitumor immunity.

Metabolic intervention represents another entry point into this ecosystem (10). Pan et al. developed a mitochondria-targeted bezafibrate derivative, Mito-BEZ, more than 200-fold more potent than the parent drug at inhibiting respiratory complexes I and III. Mito-BEZ inhibited lung tumor development in preclinical models while increasing tumor-infiltrating CD8^+^ T cells and reducing MDSCs. Notably, Mito-BEZ was reported to exert divergent metabolic effects across cellular compartments, suppressing oxidative metabolism in tumor cells while enhancing mitochondrial fitness in effector CD8^+^ T cells. This observation illustrates the potential for a single metabolic intervention to reshape both tumor-cell-intrinsic and immune arms of the microenvironment, thereby promoting an immunostimulatory state.

Macrophage-associated signatures can also be used to define the mechanisms of action of emerging anticancer strategies (11). Extending this concept, Sharma et al. showed in breast cancer models that the efficacy of the BET degrader ZBC260 required both macrophages and NK cells. BET degradation remodeled the macrophage compartment by reducing MRC1-positive immunoregulatory macrophages while enriching CD74-positive antigen-presenting macrophages, and increasing macrophage phagocytic activity. This study highlights how transcriptional and epigenetic modulation can reshape macrophage identity and function over the course of anticancer treatment.

## 3 Toward integrated reprogramming of antitumor immunity

Successful manipulation of tumor-associated myeloid cells should not be considered in isolation from the broader immune ecosystem. Reprogramming suppressive myeloid populations may relieve inhibitory pressure on antitumor immunity, but optimal therapeutic effects will likely require strategies that simultaneously strengthen the expansion, persistence, and function of immune effector cells (12). Hotchkiss et al. broadened this framework by proposing IL-7 as a next-generation adjuvant for cellular and immune therapies. Although IL-7 is not currently approved as an anticancer immunotherapy, IL-7-based treatments have been administered to more than 620 adult and pediatric patients, including multiple myeloma patients receiving CAR-T-cell therapy and patients with urothelial cancer receiving immune checkpoint inhibitors. By supporting the expansion, persistence, and functionality of CD4^+^ and CD8^+^ T cells, IL-7-based strategies may complement approaches aimed at reducing myeloid-mediated immunosuppression. Their perspective emphasizes the reciprocal nature of antitumor immunity, in which relieving suppressive myeloid constraints and strengthening effector-cell fitness represent complementary components of immune reprogramming rather than separate therapeutic objectives.

Collectively, the contributions to this Research Topic show that the biological and clinical significance of tumor-associated myeloid cells depends on their functional state, spatial organization, treatment context and temporal evolution. Progress will therefore require more precise characterization of these populations through integrated single-cell and spatial analyses, functional and metabolic profiling, and longitudinal sampling. Rather than relying solely on the identification or depletion of suppressive myeloid populations, future strategies should define when, where, and through which mechanisms individual myeloid cell states can be redirected, and which patients are most likely to benefit. Combining context-specific myeloid modulation with approaches that preserve or enhance antitumor effector-cell fitness offers a route to overcoming resistance and extending the benefit of cancer immunotherapy.
